# Longitudinal regional brain volume loss in schizophrenia: Relationship to antipsychotic medication and change in social function

**DOI:** 10.1016/j.schres.2015.06.016

**Published:** 2015-10

**Authors:** Joyce Y. Guo, Sanna Huhtaniska, Jouko Miettunen, Erika Jääskeläinen, Vesa Kiviniemi, Juha Nikkinen, Jani Moilanen, Marianne Haapea, Pirjo Mäki, Peter B. Jones, Juha Veijola, Matti Isohanni, Graham K. Murray

**Affiliations:** a Department of Psychiatry, Cambridge Biomedical Campus, University of Cambridge, Box 189 CB2 0QQ, United Kingdom; b Behavioural and Clinical Neuroscience Institute, University of Cambridge, Cambridge CB2 0SZ, United Kingdom; c Department of Psychiatry, Research Group for Clinical Neuroscience, University of Oulu, Oulu, Finland; d Department of Psychiatry, Oulu University Hospital, Oulu, Finland; e Institute of Health Sciences, University of Oulu, Oulu, Finland; f Medical Research Center Oulu, University of Oulu and Oulu University Hospital, Oulu, Finland; g Department of Diagnostic Radiology, Oulu University Hospital, Oulu, Finland; h Department of Psychiatry, Länsi-Pohja Healthcare District, Finland; i Department of Psychiatry, the Middle Ostrobothnia Central Hospital, Kiuru, Finland; j Mental Health Services, Joint Municipal Authority of Wellbeing in Raahe District, Finland; k Mental Health Services, Basic Health Care District of Kallio, Finland; l Visala Hospital, the Northern Ostrobothnia Hospital District, Finland

**Keywords:** NFBC 1966, Northern Finland Birth Cohort 1966, SIENA, Structural Image Evaluation, Using Normalisation, FSL, FMRIB software library, FLIR, FMRIB's linear image registration tool, PBVC, Percentage brain volume change, FAST, FMRIB's automated segmentation tool, TFCE, Threshold-free cluster enhancement, PANSS, Positive and Negative Syndrome Scale, CGI, Clinical Global Impression, SOFAS, Social and Occupational Functioning Assessment Scale, Longitudinal MRI, Schizophrenia, Antipsychotic medication

## Abstract

**Background:**

Progressive brain volume loss in schizophrenia has been reported in previous studies but its cause and regional distribution remains unclear. We investigated progressive regional brain reductions in schizophrenia and correlations with potential mediators.

**Method:**

Participants were drawn from the Northern Finland Birth Cohort 1966. A total of 33 schizophrenia individuals and 71 controls were MRI scanned at baseline (mean age = 34.7, SD = 0.77) and at follow-up (mean age = 43.4, SD = 0.44). Regional brain change differences and associations with clinical mediators were examined using FSL voxelwise SIENA.

**Results:**

Schizophrenia cases exhibited greater progressive brain reductions than controls, mainly in the frontal and temporal lobes. The degree of periventricular brain volume reductions were predicted by antipsychotic medication exposure at the fourth ventricular edge and by the number of days in hospital between the scans (a proxy measure of relapse duration) at the thalamic ventricular border. Decline in social and occupational functioning was associated with right supramarginal gyrus reduction.

**Conclusion:**

Our findings are consistent with the possibility that antipsychotic medication exposure and time spent in relapse partially explain progressive brain reductions in schizophrenia. However, residual confounding could also account for the findings and caution must be applied before drawing causal inferences from associations demonstrated in observational studies of modest size. Less progressive brain volume loss in schizophrenia may indicate better preserved social and occupational functions.

## 1 Introduction

Since the earliest neuroimaging studies using pneumoencephalography, it has been postulated that there is progressive brain volume reduction in schizophrenia (e.g. Moore et al., 1935; Haug, 1962). However, it was only with the application of modern neuroimaging techniques and the introduction of control groups (Johnstone et al., 1976), and in particular by the advent of longitudinal controlled MRI studies (DeLisi et al., 1995; DeLisi et al, 1997), that progressive brain changes in schizophrenia were confirmed with greater certainty. Numerous MRI studies since then have indicated that patients with schizophrenia have greater progressive volume reductions in the frontal, temporal or parietal lobes and/or progressive ventricular enlargement, compared to controls (e.g. Saijo et al., 2001; Ho et al., 2003; Nakamura et al, 2007; van Haren et al., 2007; DeLisi, 2008; van Haren et al., 2008; Andreasen et al., 2011; van Haren et al., 2011; Cobia et al., 2012). The meta-analysis by Kempton and colleagues (2010) pooled results from 13 studies examining ventricular change in a total of 473 patients and 348 controls, finding that patients show increased rates of ventricular enlargement over time compared to controls. The meta-analysis by Olabi and colleagues (2011) confirmed evidence of progressive volume loss in frontal, temporal and parietal regions, and of progressive ventricular enlargement, compared to controls. However, two other meta-analyses present differing results. One meta-analysis that focussed on gray matter changes confirmed chronic temporal lobe progressive change but suggested a nuanced picture with increased rates of widespread volume loss in the earlier stages of illness (Vita et al., 2012). A meta-analysis of progressive change in a restricted set of regions in early-onset psychosis (mean age of onset in the five included studies ranged between 13 and 16 years) suggested that the frontal lobe was the only region in which brain volume loss significantly differed between patients and controls (Fraguas et al., in press).

Given that there is significant progressive brain structural change during the course of schizophrenia, but not complete consensus even amongst meta-analyses on the regional nature of change, further studies are required to explore the detailed regional basis of structural changes. In order to evaluate the importance of morphological changes, it is critical to examine whether the progressive changes are associated with clinical and/or social function deterioration. Some groups have suggested that greater brain atrophy over time predicts poor clinical and social outcomes or greater brain reduction over time is correlated with less improvement of clinical and social outcomes and/or duration of time spent in hospital during the follow-up (e.g. DeLisi et al., 2004; Andreasen et al., 2011; Asami et al., 2012). However, the associations between regional brain structural change over time and clinical or social outcomes in schizophrenia have not been universally replicated and it remains uncertain which, if any, brain regions are more vital than others for long-term outcomes (van Haren et al., 2003; DeLisi, 2008; Olabi et al., 2011; Gutiérrez-Galve et al., 2015).

Cumulative evidence has revealed significant associations between cortical and subcortical brain structural change and antipsychotic medication exposure (e.g. Moncrieff and Leo, 2010; Ho et al., 2011; Ebdrup et al., 2013; Haijma et al., 2013; Torres et al., 2013). The systematic review by Fusar-Poli and colleagues (2013) reported that higher cumulative antipsychotic dose associated with more pronounced decrease in gray matter volume and increase in lateral ventricular size. In original studies, antipsychotic medication exposure has been associated with, for example, decreases in frontal, temporal and parietal gray matter (Ho et al., 2011), hippocampal volume (Ebdrup et al., 2011; Mamah et al., 2012) and thalamic volume (Khorram et al., 2006), and increases in the volume of the basal ganglia (Lang et al., 2004) and lateral ventricles (Crespo-Facorro et al., 2008). However, this topic is controversial; furthermore, whether typical and atypical antipsychotic medication have the same effects on regional brain structure change in patients with schizophrenia remains disputable (Ebdrup et al., 2013; Lieberman et al., 2005; McClure et al., 2006; Navari and Dazzan, 2009; Smieskova et al., 2009).

We recently reported that antipsychotic medication exposure predicted loss of total brain volume in schizophrenia over time (Veijola et al, 2014) even after controlling for symptom severity, alcohol use and weight gain; however, our recent study, like the majority of other papers on this topic, looked only at summary brain volume indices rather than a fine-grained (voxel or vertex based) level of resolution. Indeed, only a small number of voxel-based or vertex-based previous studies have examined progressive morphological change in schizophrenia at all, and only one long-term study has examined associations between medication morphological change and medication exposure at this level of resolution (van Haren et al., 2011); moreover, as that study focussed only on the cortex it could not address any association between medication exposure and ventricular change. In this study, therefore, we employed voxelwise analyses to examine progressive brain changes in schizophrenia compared to controls, and the relationship between progressive changes and symptoms, level of function and antipsychotic medication exposure.

## 2 Method

### 2.1 Participants

As described previously (Veijola et al, 2014), 33 patients with DSM-III-R (Diagnostic and Statistical Manual of Mental Disorders Third Edition Revised) schizophrenia and 71 controls, selected from a general population-based birth cohort study, the Northern Finland Birth Cohort 1966 (NFBC 1966 www.oulu.fi/nfbc), took part. Structured clinical interview for DSM-III-R (SCID) and full case records were obtained and scrutinized in order to validate the diagnoses using DSM-III-R criteria (Isohanni et al., 1997; Moilanen et al., 2003). Table 1 shows the demographic characteristics of the final 104 participants in the current study. The mean age of patients with schizophrenia was 34.1 years at baseline and 43.2 years at follow-up. The mean age of controls was 35.0 years at baseline and 43.5 years at follow-up (Table 1). There was no significant age difference between diagnostic groups. The patients who took part in the study are representative of the entire population of schizophrenia patients born in 1966 in Northern Finland in terms of age, sex and educational level (Veijola et al, 2014). The average interscan interval significantly (*t* (102) = 4.08, *p* < .000) differed between patients with schizophrenia (mean = 9.10 years, SD = 0.59) and controls (mean = 8.54 years, SD = 0.66), hence interscan interval, sex and handedness were used as covariates in the group comparison statistical analyses. Permission to gather data was obtained from the Ministry of Social and Health affairs, and the study design was approved by the Ethical Committee of the Ostrobothnian Hospital District, Oulu, Finland.

### 2.2 Clinical assessments

Clinical symptoms and social functioning in patients with schizophrenia at baseline and follow-up were examined using three clinical assessments: the Positive and Negative Syndrome Scale (PANSS), the Clinical Global Impression (CGI) and the Social and Occupational Functioning Assessment Scale (SOFAS) (Haapea et al., 2007; Veijola et al., 2014). The change score of clinical assessments were calculated by subtracting the scores of clinical assessments at follow-up from the scores of clinical assessments at baseline. We also recorded time in hospital during the interscan interval as a measure of illness severity in patients with schizophrenia, reasoning that hospitalization time was a proxy measure of relapse duration, which has been associated with volume loss in schizophrenia (Andreasen et al., 2011).

### 2.3 Antipsychotic medication exposure

Current and earlier use of antipsychotic medication was ascertained by interview for all participants at both assessments, and individual hospital and outpatient case notes were carefully reviewed to assess the use of antipsychotic medication during the follow-up. During the follow-up, three schizophrenia participants took no antipsychotic medication and three took antipsychotic medication for less than one year. We measured the use of antipsychotic medication in terms of chlorpromazine equivalents and expressed the total use of antipsychotic medication over the study period in terms of the number of dose years of 100 mg of chlorpromazine daily (Veijola et al., 2014). Dose years for total antipsychotic medication was used as a continuous variable for our primary analysis and, as secondary analyses, we examined dose years for typical and atypical antipsychotics separately.

### 2.4 The MRI scanner parameters

Brain MRI structural images from two time points in the present longitudinal study were collected by a GE Sigma 1.5 Tesla MRI scanner in Oulu University Hospital. See supplementary text for details of MRI data acquisition.

### 2.5 Regional brain change calculations

Regional brain changes in all participants were calculated using voxelwise SIENA (Structural Image Evaluation, Using Normalisation) software (Smith et al., 2002) in FSL (FMRIB software library) (Jenkinson et al., 2012). Specifically, voxelwise SIENA normalizes the images of brain edge displacements generated in SIENA, namely, flow images, into MNI152 standard space for each participant. Then, the normalized flow images of all participants are inputted into a non-parametric testing tool (Randomise; Nichols and Holmes, 2002; Hayasaka and Nichols, 2003) for statistical analyses. Notably, as voxelwise SIENA measures brain edge displacements between two time points, the results of regional analyses are purely restricted to the brain edge: SIENA differs in this respect from classical voxel-based morphometry.

### 2.6 Statistical analysis

Voxelwise statistical analyses of the brain edge displacement employed the permutation-based, voxelwise non-parametric testing tool Randomise in FSL (Nichols and Holmes, 2002; Hayasaka and Nichols, 2003). Randomise produced statistical images using the threshold-free cluster enhancement (TFCE) function, thresholded at *p* < 0.05 and corrected for multiple comparisons across the entire brain edge using family-wise error correction. See supplementary material for further details on statistical analyses.

## 3 Results

### 3.1 Regional brain structural change differences between patients with schizophrenia and controls

In unadjusted models, schizophrenia patients displayed significantly (*p* < 0.05 family-wise error corrected across the brain edge) greater regional brain reduction in widespread regions, including the bilateral frontal pole, middle and inferior frontal gyrus, parietal lobes, bilateral central gyrus, temporal lobes (including superior, middle and inferior divisions), occipital lobes, precuneous, the right lingual gyrus (mid-line occipital lobe), cerebellum and periventricular regions (supplementary Table 1 and supplementary Fig. 1).

**Fig. 1:**
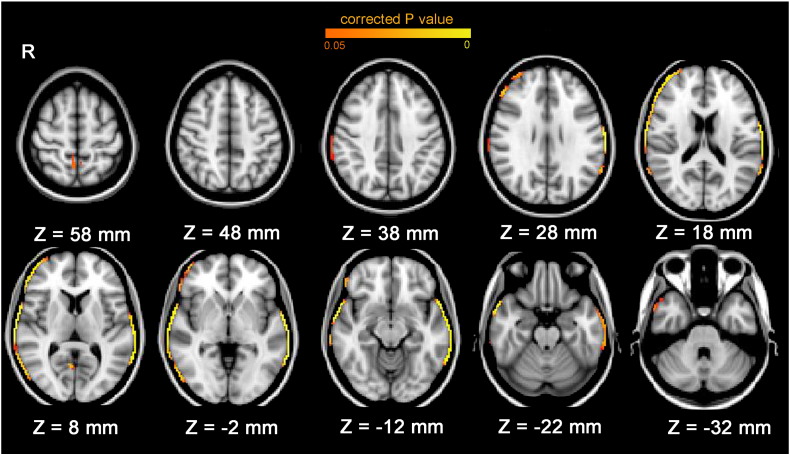
Brain edge regions with greater loss of brain volume in schizophrenia patients compared to controls (adjusted for sex, handedness and interscan interval, *p* < 0.05 family-wise error corrected across brain edge). Patients with schizophrenia showed significantly greater regional brain structural atrophy than controls in the right frontal pole, right middle and inferior frontal gyrus, bilateral central gyrus, bilateral supramarginal gyrus, bilateral temporal lobe (including superior, middle and inferior divisions), right occipital cortex inferior division, right lingual gyrus (mid-line occipital lobe) and bilateral precuneous cortex.

The results were similar when taking sex, handedness and interscan interval (follow-up time) as covariates: schizophrenia patients exhibited significantly greater regional brain reduction in the right frontal pole, right middle and inferior frontal gyrus, bilateral central gyrus, bilateral supramarginal gyrus, bilateral temporal lobe (including superior, middle and inferior divisions), right occipital cortex inferior division, right lingual gyrus (mid-line occipital lobe) and bilateral precuneous cortex (Table 2; Fig. 1). Global brain reduction was greater in patients with schizophrenia (mean = − 6.13%, SD = 2.44) than controls (mean = − 4.07%, SD = 1.43). After taking into account the percentage brain volume change (PBVC) global brain reduction (whether as a single covariate or together with other covariates), no regions showed significantly greater reduction in schizophrenia compared to controls.

Unadjusted models did not document any regions in which controls showed significantly greater brain reductions than schizophrenia patients; however, after controlling for total percentage brain volume change, sex, handedness and interscan interval (follow-up time), a significantly greater reduction in the right occipital pole was revealed in controls than in schizophrenia patients, indicating relative sparing of occipital lobe atrophy in patients (Table 3; supplementary Fig. 2).

### 3.2 Associations between regional brain structural changes and antipsychotic medication exposure in patients with schizophrenia

#### 3.2.1 Antipsychotic medication and brain reductions

**Fig. 2:**
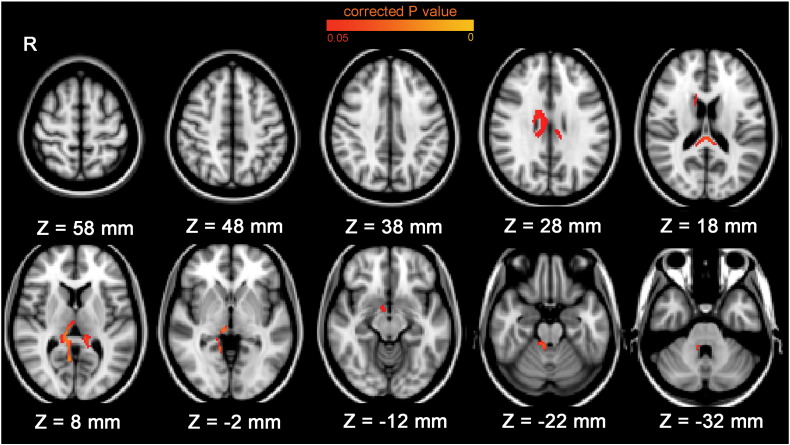
Brain edge regions associated with total antipsychotic medication exposure over time in patients with schizophrenia (uncorrected for any covariate, *p* < 0.05 family-wise error corrected across brain edge). More total antipsychotic medication exposure was significantly associated with greater regional brain reductions around the ventricles, and at the border of the substantia innominata, in schizophrenia patients.

Total antipsychotic medication exposure was significantly (*p* < 0.05 family-wise error corrected across the brain edge) associated with regional brain reductions in three clusters at the ventricular edge in patients with schizophrenia (please note that reduction in periventricular brain volume in our analyses are equivalent to ventricular enlargement) and in a cluster at the border of the substantia innominata (see Supplementary Table 2 for peak voxel coordinates). The brain areas adjacent to the areas of increased ventricular size included the bilateral lingual gyrus, right thalamus, right caudate, bilateral callosal body, right cerebellum (bordering the fourth ventricle) and the brain stem (Fig. 2). We extracted the mean brain edge movement for each of these four clusters for each participant and ran further regression analyses to determine if the associations were robust to co-varying for various potential confounders. When we adjusted for sex, interscan interval, handedness, global brain volume change and severity of illness (by including both mean PANSS and interscan hospitalization time in the models), the associations between medication and brain reductions remained significant in the cluster at the cerebellar edge bordering the fourth ventricle (unstandardized beta = − 0.007, *t* = − 3.34, *p* = 0.003) and in the cluster at the edge of the substantia innominata (unstandardized beta = − 0.002, *t* = − 2.53, *p* = 0.018).

Results of analyses examining the effects of typical and atypical medications separately revealed a similar pattern of effects in the two classes of medication; however, the effects were stronger in atypical medication analyses and only atypical medication exposure was significantly associated with brain reductions around the ventricles (Supplementary Table 3 and Supplementary Fig. 3).

### 3.3 Associations between regional brain structural changes and clinical assessments in patients with schizophrenia

#### 3.3.1 Baseline clinical assessment scores and regional brain changes

None of the baseline clinical scores predicted regional brain structural changes in patients with schizophrenia regardless of whether co-varying for PBVC or not.

#### 3.3.2 Hospitalization time and regional brain changes

The unadjusted model did not show any significant association between regional brain atrophy and hospitalization duration. However, there was a significant association between longer hospitalization duration (log10 days in hospital) during the interscan interval and ventricular enlargement at the thalamic border patients with schizophrenia, while co-varying for sex, interscan interval and handedness (Supplementary Table 4 and Supplementary Fig. 4). We extracted the mean brain edge movement for these clusters for each participant and ran further regression analyses to determine if this association was robust to co-varying for other potential confounders: global brain volume change, mean PANSS and antipsychotic medication exposure. The association between hospitalization time and brain reduction remained significant (unstandardized beta = − 0.103, *t* = − 2.40, *p* = 0.023) in the left thalamic border cluster; the association in the right thalamic border cluster was marginally significant (unstandardized beta = − 0.093, *t* = − 1.97, *p* = 0.059).

#### 3.3.3 Clinical assessment score changes and regional brain changes

Improvement in SOFAS change score was significantly associated with less reduction of the right supramarginal gyrus over time in patients with schizophrenia, co-varying for sex, handedness and interscan interval. The results remained significant after also controlling for total antipsychotic medication exposure or global brain reduction as the extra covariate (Supplementary Table 5). In unadjusted models, there was an association between frontal pole reduction and SOFAS decrease, but this was no longer significant after adjustment for sex, handedness and interscan interval. Neither CGI change scores nor PANSS change scores predicted regional brain structural changes in patients with schizophrenia regardless of co-varying for PBVC or not.

## 4 Discussion

### 4.1 Regional brain structural change differences between schizophrenia and controls

Patients with schizophrenia displayed greater significant regional brain structural reduction than controls in widespread brain regions in uncorrected analyses and when co-varying for sex, handedness and interscan interval. The results suggested that schizophrenia patients displayed excessive whole-brain reduction during the course of schizophrenia, wherein the frontal, temporal and parietal lobes exhibited non-significantly greater reductions than other brain regions, with relative sparing of the posterior occipital cortex. The current results of the frontal, temporal and parietal lobe reductions in patients replicate and extend findings from meta-analysis (Olabi et al., 2011; Vita et al., 2012). However, many previous results have stemmed from results segmenting the brain at the lobar level, whilst our results are more fine-grained, extending to the voxel level. Perhaps the most notable feature of our results is that even using a fine-grained voxel resolution technique, we document widespread cortical and periventricular loss rather than any particularly focal loss of brain volume; indeed after controlling for the overall percent volume change, there are no regions that show significantly greater reduction in schizophrenia, emphasizing how widespread the changes are.

### 4.2 Relations between regional brain structural changes in patients with schizophrenia and clinical measures

In line with some previous literature, neither clinical symptoms nor social functioning at baseline predicted regional brain structural progressive change in patients with schizophrenia (van Haren et al., 2003). A positive result would have been expected if poor functioning causes deterioration in brain volumes (for example, via inactivity). However, the lack of association should not be interpreted as excluding a causal relationship, given the modest sample size of the schizophrenia group.

Change in social and occupational function was associated with regional brain reductions in the right supramarginal gyrus (parietal lobe) in patients with schizophrenia. This association remained significant after controlling for total antipsychotic medication exposure and global brain reduction. This indicates that schizophrenia patients with greater improvement, or less deterioration, of social and occupational functions showed less regional brain reductions in the supramarginal gyrus, independent from antipsychotic medication exposure. These results may relate to existing evidence that the supramarginal gyrus is involved in neural circuits underlying the theory of mind (Fletcher et al., 1995; Gallagher et al., 2000; Carr et al., 2003; Carrington and Bailey, 2009), which is impaired in schizophrenia (Horan et al., 2012; Fiszdon et al., 2013) and is associated with social function in patients with schizophrenia (McGlade et al., 2008; Couture et al., 2011).

In unadjusted models, frontal pole reduction predicted social and occupational function decline. Evidence for structural abnormalities of the frontal pole in patients with schizophrenia have been reported in both post-mortem (Benes et al., 1991) and MRI studies (Goldstein et al., 1999; Rosso et al., 2010; Byun et al., 2012), with relationships to impaired executive functions (Orellana and Slachevsky, 2013) in schizophrenia patients and social cognition (Bludau et al., 2014) in general. As this finding did not remain significant after adjustment for sex, handedness and interscan interval, we are cautious in interpreting its significance. It should be noted that taking MRI and clinical measures at two arbitrary time points and examining associations between their changes is a crude method with which to investigate relationships between biology and course, as the clinical severity of schizophrenia may fluctuate markedly as the illness waxes and wanes,

We found an association between days spent in hospital and ventricular enlargement around the thalamic border. This measure is a proxy for time spent in relapse. As the association remained after controlling for medication exposure and other potential confounders, this result is in keeping with the result of the Suffolk County New York 10-year follow-up study, which showed that total days in hospital was associated with left ventricular progressive enlargement between 5 and 10 years post-onset (DeLisi et al, 2004), and with the results of the Iowa study that linked loss of brain volume to time spent in relapse, although the location of their association was in the frontal lobe (Andreasen et al., 2013). This result is consistent with the possibility that time spent in psychotic relapse may be causally related to brain volume loss, although causality cannot be inferred from an observational study such as ours.

### 4.3 Antipsychotic medication exposure related regional brain structural change in patients with schizophrenia

The present study showed that antipsychotic medication exposure was associated with greater brain reduction around the ventricles (i.e. ventricular enlargement) in patients with schizophrenia, independent from illness severity and time in hospital. These results are consistent with an early speculation by the neurologist Marsden (1976) and replicate a milestone study with the largest sample size of patients with schizophrenia (*N* = 211) so far and with a long follow-up period (7.2 years on average compared to 9.1 years in our study) (Ho et al., 2011). Although, a previous meta-analysis failed to document an association between antipsychotic medication exposure and progressive ventricular enlargement in schizophrenia (Fusar-Poli et al., 2013), we note that our study is the first long-term study to examine the association between ventricular or periventricular changes over time and medication exposure at a voxel-level of resolution. Indeed our study is only the second long-term study to examine the association between brain changes and medication exposure at the level of resolution of the voxel or vertex, and the only previous study, by Van Haren and colleagues (van Haren et al., 2011), examined only cortical thickness, and thus did not assess the ventricles or periventricular regions.

There were no marked differences in the effects of typical and atypical medications, although our modest sample size has limited power to detect differential effects of different medications, and many patients had taken different medications at different times before and during the follow-up, making it hard to completely segregate the effects of different medications. We also noted a relationship between reduction at the inferior edge of the substantia innominata and medication exposure. Other studies have suggested that association medication and brain volume reduction is likely to be more general (Ho et al., 2011; van Haren et al., 2011; Fusar-Poli et al., 2013; Veijola et al., 2014). However, whilst an advantage of a voxel-based study such as this is regional specificity, the correction for multiple comparisons necessitated may result in type II error; we were unable to document statistically robust regional effects of medication that survived stringent correction for multiple comparisons across other regions. We note that others have argued that the ventricular enlargement of schizophrenia patients in previous studies is not directly associated with brain reductions in the adjacent focal subcortical regions/white matter, but rather may indicate a (global) gray matter decrease in schizophrenia patients (DeLisi et al., 2006; Horga et al., 2011).

### 4.4 Strengths and limitations

A significant strength of the current longitudinal study is our design that ensured the same chronological age at baseline time for all participants, which reduces noise that could otherwise have been introduced in the by non-linear ageing effects, and removes the necessity to control for age in our study. Another advantage of the current study was the use of the FSL software tool SIENA, which has been argued to be the most robust software for estimating brain volume change over time and can compensate for upgrades in MRI scanner hardware and software (de Bresser et al., 2011). There were several limitations to be noticed in the current study, which included an updated MRI scanner over the follow-up; a modest sample size of patients with schizophrenia; different criteria for PANSS scoring at two time points, and, as in nearly all observational medical studies, imperfect monitoring of medication exposure during follow-up, although we painstakingly scrutinized hospital records to minimise measurement error as much as possible. In future work, the use of ingestible medication event markers or repeated blood monitoring of drug levels would be an advantage although sustaining these procedures over several years will be challenging. Whilst our study adds to evidence of association between antipsychotic medication exposure and brain morphological change, observational studies such as this one cannot establish causality. In spite of our careful attempts to control for various factors, it remains possible that the associations we observed could be secondary to residual confounding. A common response by the clinician to a severely ill patient is to add more medicine: this is understandable under the common medical philosophy that “more is better” (Malhotra et al., 2015). Severity may be confounded with treatment dose in a way that makes it very hard (in observational studies) to interpret whether any long-term brain changes are ultimately related to medication or are secondary to a more severe illness type, or some mixture of the two or to other potential causal factors yet to be identified. In the future, observational studies of the effects of antipsychotic (dopamine receptor antagonist) medication on brain structure in other disorders where their use is increasingly common (e.g. Tourette’s syndrome, bipolar disorder, depression), and additional controlled trials in patients with schizophrenia and in experimental animals using brain structure as an outcome measure, will help interpret the results of schizophrenia observational studies.

### 4.5 Conclusions

Our study documents excessive volume loss in schizophrenia compared to controls in widespread regions. However, as volume loss had few associations with clinical and functional outcomes, its significance is difficult to judge. The findings of associations between progressive loss of brain volume and antipsychotic medication, and with time spent in relapse, merit further study. The association between supramarginal gyrus volume loss and decrease in social and occupational performance is not secondary to medication, and emphasizes the importance of parietal lobe structure in social function in schizophrenia.

## Role of the funding source

The funders had no role in study design, data collection and analysis, decision to publish or preparation of the manuscript.

## Contributors

Conceived and designed the experiments: Juha Veijola, Jouko Miettunen, Erika Jääskeläinen, Vesa Kiviniemi, Peter B. Jones, Matti Isohanni, Graham K. Murray. Performed the experiments: Juha Veijola, Jani S. Moilanen, Erika Jääskeläinen, Pirjo Mäki, Vesa Kiviniemi and Juha Nikkinen.

Analyzed the data: Juha Veijola, Joyce Y. Guo, Sanna Huhtaniska, Vesa Kiviniemi, Juha Nikkinen, Marianne Haapea and Graham K. Murray.

Wrote the paper: Joyce Y. Guo, Sanna Huhtaniska, Jouko Miettunen, Erika Jääskeläinen, Vesa Kiviniemi, Juha Nikkinen, Jani S. Moilanen, Marianne Haapea, Pirjo Mäki, Peter B. Jones, Juha Veijola, Matti Isohanni and Graham K. Murray.

## Conflicts of interest

All authors report no conflicts of interest.

## Figures and Tables

**Table 1:** Demographic data.

	Schizophrenia			Controls		
Mean	SD	*N*	Mean	SD	*N*
Age at time point one (years)	34.12	0.63	33	34.97	0.67	71
Age at time point two (years)	43.21	0.43	43.52	0.41
Follow-up time (years)	9.10	0.59	8.54	0.66
Gender (men/women)	19/14		43/28	
Handedness (right/left)	31/2		66/5	

**Table 2:** Regions in which there were voxels showing greater volume loss in schizophrenia patients compared to controls (*p* < 0.05, FWE corrected, adjusted for sex, handedness and interscan interval).

Regions	Group	Mean of Edge Displacement (mm)		Number of Voxels	*t*	*p* (peak)	Coordinate of *p* Peak Voxel (MNI152 mm)		
Mean	SD	*x*	*y*	*z*
Right frontal pole	Schizophrenia	− 0.42	0.29	694	4.20	.004	48	52	12
Control	− 0.21	0.17
Right middle frontal gyrus	Schizophrenia	− 0.43	0.30	169	3.72	.006	56	34	18
Control	− 0.20	0.22
Right inferior frontal gyrus	Schizophrenia	− 0.43	0.23	330	3.58	.004	64	6	2
Control	− 0.25	0.17
Bilateral central gyrus	Schizophrenia	− 0.42	0.23	637	4.58	< .001	66	0	2
Control	− 0.22	0.14
Bilateral supramarginal gyrus	Schizophrenia	− 0.35	0.26	607	5.65	< .001	− 70	− 44	− 2
Control	− 0.14	0.15
Bilateral temporal lobe	Schizophrenia	− 0.29	0.18	1726	5.67	< .001	− 68	− 46	− 10
Control	− 0.11	0.10
Right occipital cortex (inferior division)	Schizophrenia	− 0.28	0.23	182	3.57	.010	60	− 70	2
Control	− 0.09	0.24
Right lingual gyrus (mid-line occipital)	Schizophrenia	− 0.04	0.08	34	6.14	.011	8	− 62	6
Control	0.05	0.07
Bilateral precuneous cortex	Schizophrenia	− 0.13	0.10	95	4.68	.026	− 4	− 58	54
Control	− 0.04	0.05

Note: For reporting purposes, brain changes (*p* < 0.05 family-wise error corrected across the brain edge) were parcellated into regions using the Harvard–Oxford cortical structural atlas/MNI structural atlas.

**Table 3:** Regions showing greater volume loss in controls compared to schizophrenia patients (*p* < 0.05 FWE corrected, adjusted for sex, handedness, interscan interval and percentage brain volume change).

Clusters	Group	Mean of Edge Displacement (mm)		Number of Voxels	*t*	*p* (peak)	Coordinate of *p* Peak Voxel (MNI152 mm)		
Mean	SD	*x*	*y*	*z*
Right occipital pole	Schizophrenia	− 0.28	0.57	105	4.38	.021	18	− 102	− 12
Control	− 0.42	0.48
