# Supplementary material for: Longitudinal regional brain volume loss in schizophrenia: Relationship to antipsychotic medication and change in social function
Source: Schizophr Res. 2015 Oct;168(1-2):297–304. doi: 10.1016/j.schres.2015.06.016 (PMC4604250; doi:10.1016/j.schres.2015.06.016)
Supplement: Supplementary file 1 — Supplementary materials. [file mmc1.docx]

# Supplementary material: Longitudinal regional brain volume loss in schizophrenia: relationship to antipsychotic medication and change in social function

Joyce Y. Guo^1,2^, Sanna Huhtaniska^3,6^, Jouko Miettunen^3,5,6^, Erika Jääskeläinen^5,6^, Vesa Kiviniemi^7^, Juha Nikkinen^7^, Jani S. Moilanen^3^, Marianne Haapea^7^ , Pirjo Mäki^3,4, 8^, Peter B. Jones^1^, Juha Veijola^3,4^, Matti Isohanni^3,4^, Graham K. Murray^1,2^

^1^ Department of Psychiatry, University of Cambridge, Box 189 Cambridge Biomedical Campus, CB2 0QQ, United Kingdom

^2^ Behavioural and Clinical Neuroscience Institute, University of Cambridge, Cambridge CB2 2QQ, UK.

^3^ Department of Psychiatry, Institute of Clinical Medicine, University of Oulu, Oulu, Finland.

^4^ Department of Psychiatry, Oulu University Hospital, Oulu, Finland.

^5^Institute of Health Sciences, University of Oulu, Oulu, Finland.

^6^Medical Research Center Oulu, University of Oulu and Oulu University Hospital, Oulu, Finland.

^7^Department of Diagnostic Radiology, Oulu University Hospital, Oulu, Finland.

^8^ Department of Psychiatry, Länsi-Pohja healthcare district, Finland; Department of Psychiatry, the Middle Ostrobothnia Central Hospital, Kiuru, Finland; Mental health services, Joint Municipal Authority of Wellbeing in Raahe District, Finland; Mental health services, Basic Health Care District of Kallio, Finland; and Visala Hospital, the Northern Ostrobothnia Hospital District, Finland.

### Supplementary hypotheses and methods

In the present longitudinal study we assessed regional brain structural changes and its association with clinical outcomes and antipsychotic medication exposure using voxelwise analyses. Specifically, we hypothesized schizophrenia patients would show greater regional brain reductions mainly in the frontal and temporal lobe compared to controls; there would be significant correlations between structural changes and changes in clinical symptoms or social functioning over time in patients with schizophrenia; the lower scores of clinical symptoms and social functioning at baseline would predict more fronto-temporal brain structural reductions over time in patients with schizophrenia; there would be significant associations between antipsychotic medication exposure and widespread regional brain reductions in patients with schizophrenia over time; finally, antipsychotic medication class (typical and atypical) would have different effects on regional brain changes in schizophrenia patients. In addition, as suggested by an anonymous referee, we examined the association between time spent in hospital and brain changes, as it has been suggested that time spent in relapse predicts (possibly through a causal process) brain volume loss in schizophrenia.

We applied our hypotheses on a psychotic subsample of the Northern Finland Birth Cohort 1966. This database is a birth cohort population based longitudinal study, which is available to prospective researchers ([www.oulu.fi/nfbc](http://www.oulu.fi/nfbc)). To explore regional brain changes over time, we acquired the T1-weighted images at baseline using a three dimensional spoiled gradient echo (3D SPGR) sequence (slice thickness = 1.5 mm; in-plane resolution matrix size 256 x 256; voxel size 1.5 mm x 1 mm x 1 mm; TR = 35 ms; TE = 5 ms; and flip angle = 35^◦^). Prior to the second time point the scanner was up-graded into HDxt with a new gradient system and an 8 channel receiving coil. The T1-weighted images at follow-up were acquired using a 3D fast spoiled gradient echo (3D FSPGR) sequence (slice thickness = 1 mm; in-plane resolution matrix size 256 x 256; voxel size 1 mm x 1 mm x 1 mm; repetition time 12.576 ms; echo time 5.3 ms; and flip angle = 20^◦^). For the purpose of reporting the results in tables, the voxel-based maps of significant areas of change were reported with the aid of anatomical atlases supplied with FSL (the Harvard-Oxford cortical Structural Atlas/MNI Structural Atlas for cortical regions and Harvard-Oxford Subcortical Structural Atlas for subcortical regions). We take into account global change by covarying for total percent brain volume change (PBVC). We also present case control analyses adjusted for sex, handedness and interscan interval (follow-up time), and within the schizophrenia sample we present the results of association between brain volume change and medication exposure, controlling for time spent in hospital. All results are family-wise error corrected for multiple comparisons unless otherwise stated.

### Supplementary results

#### Typical antipsychotic medication exposure

There was no significant association between typical antipsychotic medication exposure and regional brain reductions in patients with schizophrenia regardless of whether controlling for any covariates.

#### Atypical antipsychotic medication exposure

The uncorrected model revealed a significant association between greater atypical antipsychotic medication exposure and greater brain reductions around the ventricles in adjacent regions/: the right lingual gyrus, right thalamus and the bilateral callosal body (Supplementary Table 3 and Supplementary Figure 3).

### Supplementary discussion

In contrast with previous findings, the current study illustrated that only atypical antipsychotic medication exposure was associated with brain reduction around the ventricles in patients with schizophrenia, while taking various confounding factors as covariates. Notably, typical antipsychotic medications exposure was more common than atypical antipsychotic medication exposure at baseline in the current study, whereas the use of atypical antipsychotics was more common than of typical antipsychotics during the 9-year follow-up ([Husa et al., 2014](#_ENREF_2)). Given the modest sample size, the current results may reflect insufficient statistical power to reveal an association between typical antipsychotic medication exposure and regional brain structural changes. Indeed, in our study, the direction of the effect of typical and atypical medication was similar, although the particular effect sizes varied.

Based on longitudinal studies in recent years, there is mixed evidence for differential effects of the two types of antipsychotic medication exposure on brain morphological change in patients with schizophrenia, with some studies failing to observe differential medication effects on brain change. ([Mattai et al., 2010](#_ENREF_6); [Roiz-Santianez et al., 2012](#_ENREF_7)). An influential randomized double-blind study investigated the different effects of typical antipsychotic medication (haloperidol (2–20 mg/d)) and atypical antipsychotic medication (olanzapine (5–20 mg/d)) on brain structural change in various stages of patients with schizophrenia. The study revealed patients treated with haloperidol showed the greater total and frontal gray matter volume loss than patients treated with olanzapine during the first 12 weeks follow-up. However, such significantly different effects of typical and atypical antipsychotic medication exposure on brain structural change were no longer significant at 52 and 104 weeks follow-up ([Lieberman et al., 2005](#_ENREF_5)). With cumulative clinical evidence supporting that equivalent treatment effect of two types of antipsychotic medication on positive symptoms ([Geddes et al., 2000](#_ENREF_1)), cognitive impairments ([Keefe et al., 2007](#_ENREF_4)) and social functioning (e.g. quality of life ([Jones et al., 2006](#_ENREF_3))), it is possible that these two classes of medication may have similar effects on brain structural change, though we recognize the possibility that individuals medications may vary in their propensity to induce brain structural change. The exact mechanism of typical and atypical antipsychotic medication on cortical and subcortical brain structure should be explored in greater detail in further clinical and preclinical studies with sufficient power to examine whether this is a medication class effect or if there is variability between medications in their effects on brain structure over time.

### Supplementary References

Geddes, J., Freemantle, N., Harrison, P., Bebbington, P., 2000. Atypical antipsychotics in the treatment of schizophrenia: systematic overview and meta-regression analysis. BMJ 321, 1371-1376.

Husa, A.P., Rannikko, I., Moilanen, J., Haapea, M., Murray, G.K., Barnett, J., Jones, P.B., Isohanni, M., Koponen, H., Miettunen, J., Jaaskelainen, E., 2014. Lifetime use of antipsychotic medication and its relation to change of verbal learning and memory in midlife schizophrenia - An observational 9-year follow-up study. Schizophr Res 158, 134-141.

Jones, P.B., Barnes, T.R., Davies, L., Dunn, G., Lloyd, H., Hayhurst, K.P., Murray, R.M., Markwick, A., Lewis, S.W., 2006. Randomized controlled trial of the effect on Quality of Life of second- vs first-generation antipsychotic drugs in schizophrenia: Cost Utility of the Latest Antipsychotic Drugs in Schizophrenia Study (CUtLASS 1). Arch Gen Psychiatry 63, 1079-1087.

Keefe, R.S., Bilder, R.M., Davis, S.M., Harvey, P.D., Palmer, B.W., Gold, J.M., Meltzer, H.Y., Green, M.F., Capuano, G., Stroup, T.S., McEvoy, J.P., Swartz, M.S., Rosenheck, R.A., Perkins, D.O., Davis, C.E., Hsiao, J.K., Lieberman, J.A., 2007. Neurocognitive effects of antipsychotic medications in patients with chronic schizophrenia in the CATIE Trial. Arch Gen Psychiatry 64, 633-647.

Lieberman, J.A., Tollefson, G.D., Charles, C., Zipursky, R., Sharma, T., Kahn, R.S., Keefe, R.S., Green, A.I., Gur, R.E., McEvoy, J., Perkins, D., Hamer, R.M., Gu, H., Tohen, M., 2005. Antipsychotic drug effects on brain morphology in first-episode psychosis. Arch Gen Psychiatry 62, 361-370.

Mattai, A., Chavez, A., Greenstein, D., Clasen, L., Bakalar, J., Stidd, R., Rapoport, J., Gogtay, N., 2010. Effects of clozapine and olanzapine on cortical thickness in childhood-onset schizophrenia. Schizophr Res 116, 44-48.

Roiz-Santianez, R., Tordesillas-Gutierrez, D., Ortiz-Garcia de la Foz, V., Ayesa-Arriola, R., Gutierrez, A., Tabares-Seisdedos, R., Vazquez-Barquero, J.L., Crespo-Facorro, B., 2012. Effect of antipsychotic drugs on cortical thickness. A randomized controlled one-year follow-up study of haloperidol, risperidone and olanzapine. Schizophr Res 141, 22-28.

### Supplementary tables

Supplementary Table 1: Regions showing greater volume loss in schizophrenia patients compared to controls (unadjusted model)

| Regions^a^ | Group | Mean of edge displacement (mm) | | Number of voxel | t | p (peak) | Coordinate of peak voxel (MNI152mm) | | |
| --- | --- | --- | --- | --- | --- | --- | --- | --- | --- |
|  |  | Mean | SD |  |  |  | x | y | z |
| Bilateral frontal pole | Schizophrenia | -0.40 | 0.23 | 2376 | 4.27 | <.001 | 48 | 52 | 8 |
|  | Control | -0.23 | 0.14 |  |  |  |  |  |  |
| Bilateral middle frontal gyrus | Schizophrenia | -0.41 | 0.27 | 375 | 4.34 | <.001 | 58 | 32 | 16 |
|  | Control | -0.22 | 0.20 |  |  |  |  |  |  |
| Bilateral inferior frontal gyrus | Schizophrenia | -0.42 | 0.20 | 787 | 4.22 | <.001 | 58 | 30 | 16 |
|  | Control | -0.27 | 0.14 |  |  |  |  |  |  |
| Bilateral central gyrus | Schizophrenia | -0.39 | 0.22 | 1125 | 4.82 | <.001 | 70 | -14 | 10 |
|  | Control | -0.22 | 0.14 |  |  |  |  |  |  |
| Bilateral parietal lobe | Schizophrenia | -0.28 | 0.17 | 2553 | 4.81 | <.001 | 70 | -8 | 10 |
|  | Control | -0.12 | 0.10 |  |  |  |  |  |  |
| Bilateral temporal lobe | Schizophrenia | -0.28 | 0.16 | 2351 | 5.84 | <.001 | -68 | -44 | -12 |
|  | Control | -0.11 | 0.10 |  |  |  |  |  |  |
| Bilateral occipital lobe | Schizophrenia | -0.34 | 0.22 | 644 | 4.11 | <.001 | -68 | -54 | 8 |
|  | Control | -0.16 | 0.21 |  |  |  |  |  |  |
| Cerebellum | Schizophrenia | -0.37 | 0.22 | 97 | 3.26 | .016 | -54 | -64 | -28 |
|  | Control | -0.23 | 0.18 |  |  |  |  |  |  |
| Bilateral periventricles | Schizophrenia | -0.32 | 0.39 | 821 | 4.26 | .009 | -12 | -38 | 8 |
|  | Control | -0.14 | 0.15 |  |  |  |  |  |  |
| Right lingual gyrus (mid-line occipital) | Schizophrenia | -0.09 | 0.16 | 93 | 6.14 | .009 | 8 | -62 | 6 |
|  | Control | 0.02 | 0.06 |  |  |  |  |  |  |
| Bilateral precuneous cortex | Schizophrenia | -0.15 | 0.10 | 318 | 5.03 | .012 | 6 | -50 | 60 |
|  | Control | -0.05 | 0.06 |  |  |  |  |  |  |

^a^ Areas with voxels that show significant differences between groups (p < 0.05 family-wise error corrected across the brain edge). For the purposes of reporting, the results were parcellated into regions using the Harvard-Oxford cortical Structural Atlas/MNI Structural Atlas.

**Supplementary Table 2: Regional brain volume reductions (p<0.05 FWE corrected) associated with total antipsychotic medication exposure (unadjusted model)**

| Clusters | Number of voxels | t | p (peak) | Coordinate of peak voxel (MNI152mm) | | |
| --- | --- | --- | --- | --- | --- | --- |
|  |  |  |  | x | y | z |
| Right substantia innominata | 21 | 3.91 | .045 | 6 | -2 | -12 |
| Cerebellar border with 4^th^ ventricle | 80 | 4.46 | .030 | 10 | -42 | -28 |
| Callosum splenium ventricular border | 199 | 3.35 | .042 | 14 | -12 | 30 |
| Thalamic ventricular border | 559 | 4.45 | .020 | 12 | -50 | 2 |

Supplementary Table 3: Regional periventricular changes (p<0.05 FWE corrected) associated with atypical antipsychotic medication exposure (unadjusted model)

| Clusters | Number of voxels | t | p (peak) | Coordinate of peak voxel (MNI152mm) | | |
| --- | --- | --- | --- | --- | --- | --- |
|  |  |  |  | x | y | z |
| Callosum splenium ventricular border | 340 | 3.95 | .031 | 12 | -48 | 4 |

**Supplementary Table 4: Regional brain volume reductions associated with hospitalisation time***

| Clusters | Number of voxels | t | p (peak) | Coordinate of peak voxel (MNI152mm) | | |
| --- | --- | --- | --- | --- | --- | --- |
|  |  |  |  | x | y | z |
| Left thalamus | 134 | 3.31 | .041 | -32 | -38 | -2 |
| Right thalamus | 38 | 3.32 | .047 | 12 | -28 | 6 |

*No regions were significant in the unadjusted model: these clusters of voxels in the bilateral thalamus were significant in a model adjusted for gender, interscan interval and handedness.

**Supplementary Table 5: Regional brain volume changes (p<0.05 FWE corrected) associated with change in SOFAS**

| Model | Region | Number of voxel | t | Corrected p (peak) | Coordinate of peak voxel (MNI152mm) | | |
| --- | --- | --- | --- | --- | --- | --- | --- |
|  |  |  |  |  | x | y | z |
| No covariate | Left frontal pole | 20 | 3.94 | .045 | -10 | 66 | -18 |
| Only total medication | Bilateral frontal pole | 161 | 4.00 | .038 | -12 | 66 | -16 |
| Only percentage brain volume change | Left frontal pole | 5 | 3.93 | .049 | -36 | 62 | -6 |
| Sex, handedness and interscan interval | Right supramarginal gyrus | 35 | 4.85 | .034 | 70 | -38 | 22 |
| Sex, handedness, interscan interval and total medication | Right supramarginal gyrus | 21 | 4.70 | .040 | 70 | -38 | 24 |
| Sex, handedness, interscan interval and percentage brain volume change | Right supramarginal gyrus | 41 | 4.74 | .034 | 70 | -36 | 20 |

### Supplementary figures


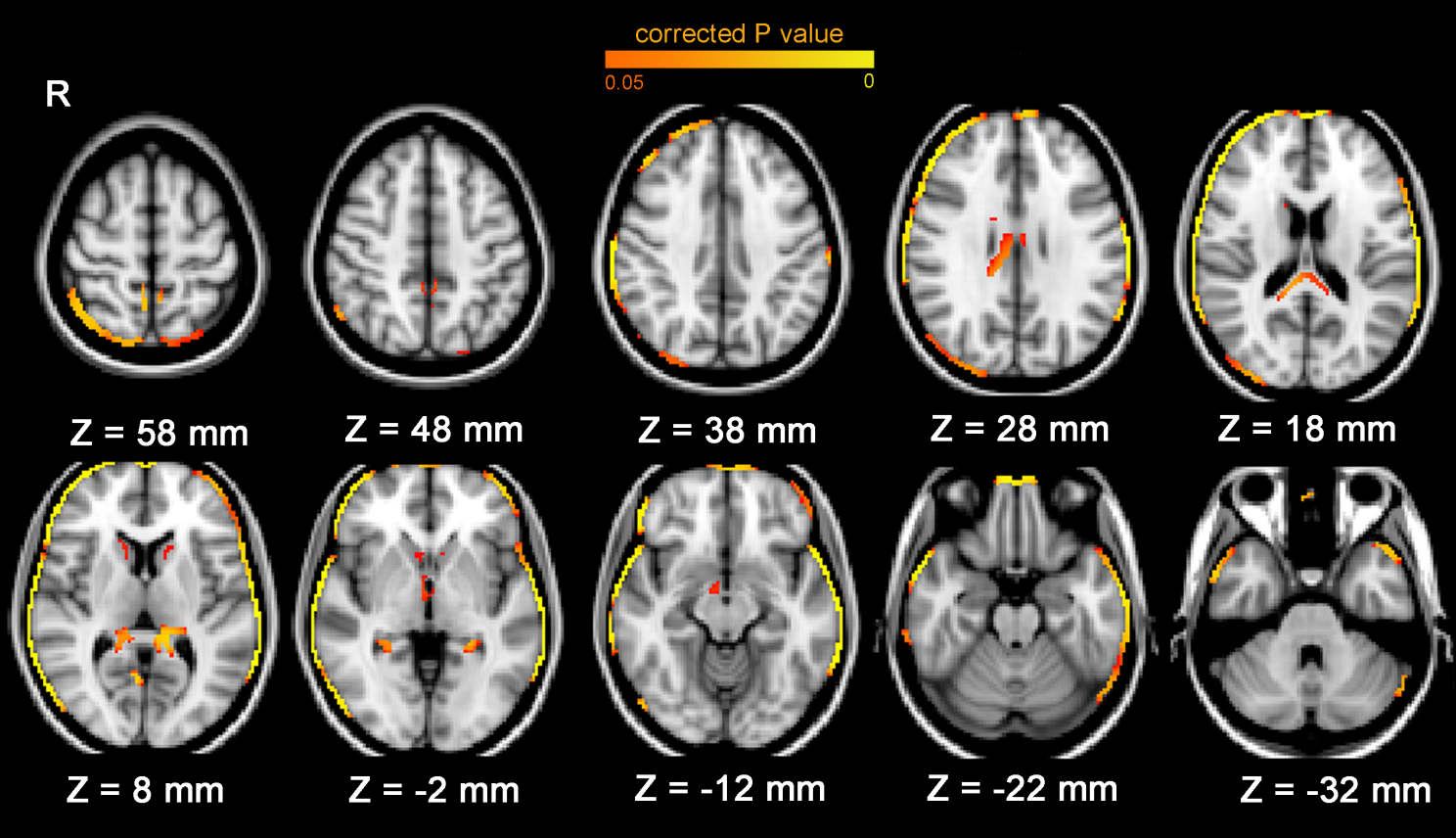


Supplementary Figure 1: Regions with greater loss of brain volume in schizophrenia patients compared to controls (unadjusted model, p < 0.05 family-wise error corrected across brain edge). Patients with schizophrenia exhibited significantly greater regional brain atrophy than controls in widespread brain regions.


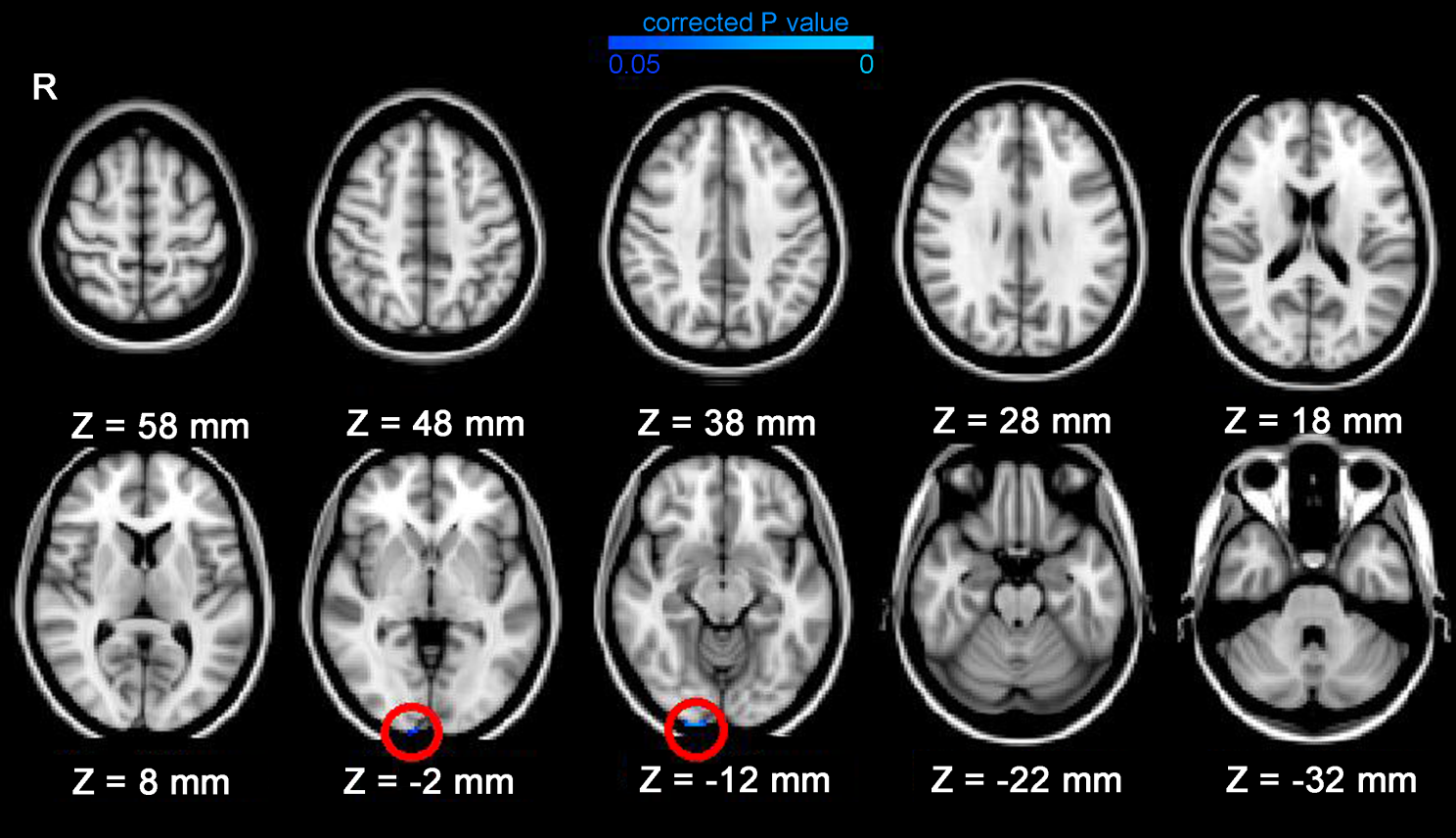


Supplementary Figure 2: Regions with greater loss of brain volume in controls compared to schizophrenia patients (adjusted for sex, handedness, interscan interval and percentage brain volume change, p < 0.05 family-wise error corrected across brain edge). Controls exhibited significantly greater regional brain reduction than patients with schizophrenia in the right occipital pole.


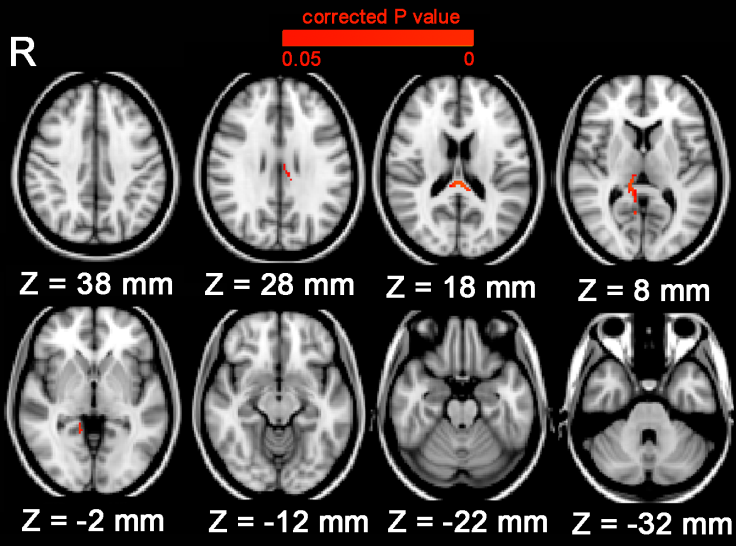


Supplementary Figure 3: Regions associated with atypical antipsychotic medication exposure over time in patients with schizophrenia (unadjusted model, p < 0.05 family-wise error corrected across brain edge).


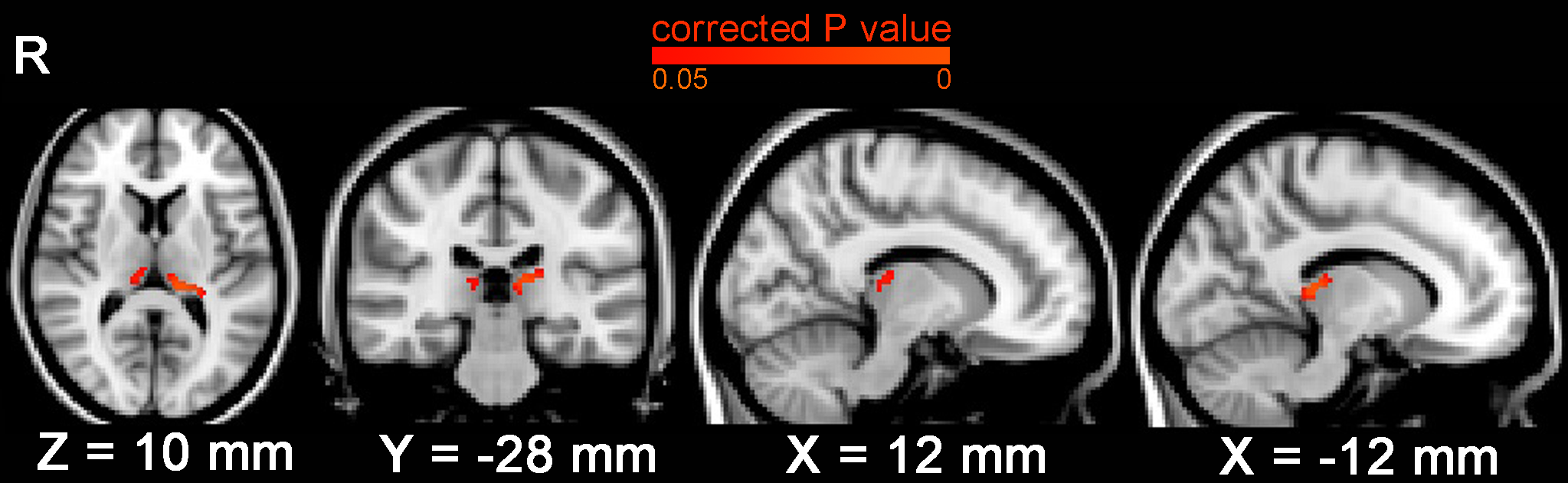


Supplementary Figure 4: Regions associated with longer hospitalization duration in patients with schizophrenia (adjusted for sex, interscan interval and handedness, p < 0.05 family-wise error corrected across brain edge).


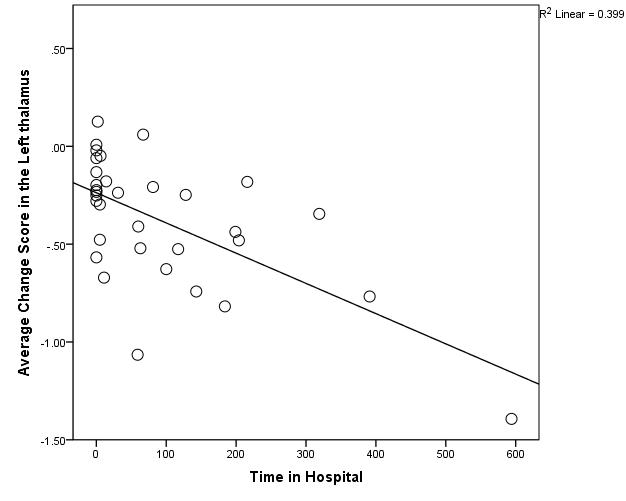


Supplementary Figure 5: Scatterplot of the association between days in hospital and the average change score (edge movement in mm) of the voxels in the left thalamic cluster determined by FSL analyses.


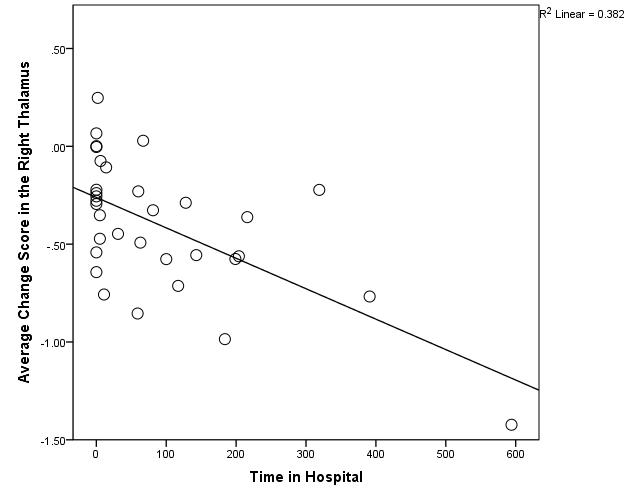


Supplementary Figure 6: Scatterplot of the association between days in hospital and the average change score (edge movement in mm) of the voxels in the right thalamic cluster determined by FSL analyses.
